# Optimizing Nitrogen Fertilization to Enhance Productivity and Profitability of Upland Rice Using CSM–CERES–Rice

**DOI:** 10.3390/plants12213685

**Published:** 2023-10-25

**Authors:** Tajamul Hussain, David J. Mulla, Nurda Hussain, Ruijun Qin, Muhammad Tahir, Ke Liu, Matthew T. Harrison, Sutinee Sinutok, Saowapa Duangpan

**Affiliations:** 1 Agricultural Innovation and Management Division, Faculty of Natural Resources, Prince of Songkla University, Hat Yai 90112, Songkhla, Thailand or hussaita@oregonstate.edu (T.H.);; 2 Hermiston Agricultural Research and Extension Center, Oregon State University, Hermiston, OR 97838, USA; 3 Department of Soil, Water, and Climate, University of Minnesota, 506 Borlaug Hall, 1991 Upper Buford Circle, St. Paul, MN 55108, USA; 4 Tasmanian Institute of Agriculture, University of Tasmania, Newnham Drive, Launceston, TAS 7248, Australia; 5 Coastal Oceanography and Climate Change Research Center, Prince of Songkla University, Hat Yai 90110, Songkhla, Thailand; 6 Faculty of Environmental Management, Prince of Songkla University, Hat Yai 90110, Songkhla, Thailand

**Keywords:** DSSAT, nitrogen uptake, grain yield, harvest index, marginal net return

## Abstract

Nitrogen (N) deficiency can limit rice productivity, whereas the over- and underapplication of N results in agronomic and economic losses. Process-based crop models are useful tools and could assist in optimizing N management, enhancing the production efficiency and profitability of upland rice production systems. The study evaluated the ability of CSM–CERES–Rice to determine optimal N fertilization rate for different sowing dates of upland rice. Field experimental data from two growing seasons (2018–2019 and 2019–2020) were used to simulate rice responses to four N fertilization rates (N_30_, N_60_, N_90_ and a control–N_0_) applied under three different sowing windows (SD1, SD2 and SD3). Cultivar coefficients were calibrated with data from N_90_ under all sowing windows in both seasons and the remaining treatments were used for model validation. Following model validation, simulations were extended up to N_240_ to identify the sowing date’s specific economic optimum N fertilization rate (EONFR). Results indicated that CSM–CERES–Rice performed well both in calibration and validation, in simulating rice performance under different N fertilization rates. The *d*-index and nRMSE values for grain yield (0.90 and 16%), aboveground dry matter (0.93 and 13%), harvest index (0.86 and 7%), grain N contents (0.95 and 18%), total crop N uptake (0.97 and 15%) and N use efficiencies (0.94–0.97 and 11–15%) during model validation indicated good agreement between simulated and observed data. Extended simulations indicated that upland rice yield was responsive to N fertilization up to 180 kg N ha^−1^ (N_180_), where the yield plateau was observed. Fertilization rates of 140, 170 and 130 kg N ha^−1^ were identified as the EONFR for SD1, SD2 and SD3, respectively, based on the computed profitability, marginal net returns and N utilization. The model results suggested that N fertilization rate should be adjusted for different sowing windows rather than recommending a uniform N rate across sowing windows. In summary, CSM–CERES–Rice can be used as a decision support tool for determining EONFR for seasonal sowing windows to maximize the productivity and profitability of upland rice production.

## 1. Introduction

Rice is one of the major cereal crops significantly contributing to food security [1]. To meet the demand, rice production is continuously increasing [2]. Thailand is one of the major rice-producing countries and rice is the country’s major cereal produced on large scales to meet local food demand. According to FAO [1], Thailand is the second largest country in Southeast Asia, whereas major rice-producing areas in Thailand are in the Northern, Central and Northeastern regions. Approximately 6% of the total rice cultivation is contributed by Southern Thailand [3]. Due to an increase in demand, however, local rice production is not sufficient and rice is supplied from other parts of the country to Southern Thailand [4]. Upland rice in Southern Thailand is grown during the rainy season [5] by small land holders as a sole crop or intercrop, with young rubber and other tree plantations. Upland rice production is vulnerable due to a lack of sufficient research evidence related to nutrient management and the traditional agronomic management practices employed by farmers. In addition, climate change has triggered variations in seasonal weather patterns [6], impacting the rainfed upland rice production. In this scenario, it becomes critical to investigate and adopt updated agronomic management practices, i.e., optimal nutrient application according to the sowing period to enhance the efficiency of upland rice production systems. 

Rice cropping systems are generally deficient in nitrogen (N) [7,8], which is a critical element in rice growth. Rice crop productivity is in turn affected by this deficiency of N as it is a critical nutrient [9] for rice growth. The application of the improper dose of N results in a range of consequences in rice production, including agronomic and economic losses, i.e., the underapplication of N results in the poor rice performance [4], whereas overapplication results in an increased pest infestation and crop lodging, and impacts quality as well as the quantity of rice produced [9]. Hence, it is necessary to apply a suitable dose of N that can sustain rice productivity. Considering the recommendations of N fertilizer application for upland rice in Thailand, there has not been any recommendation based on the adopted sowing windows, even though a wide sowing window prevails for upland rice production in Thailand. The prevalence of a wide sowing window for rainfed upland rice is due to variations in the onset of the rainy season in Thailand, which occurs between May and October [10]. Farmers sow upland rice during this period in response to soil moisture availability [4]. Similarly, a range of N fertilization rate is practiced using urea as a fertilizer source by farmers as they are concerned with attaining higher yields. A fertilization rate from 34 to 69 kg N ha^−1^ was recommended based on various rice cultivars by the Rice Department of Thailand [11]. Another recommendation of 49–82 kg N ha^−1^ was made based on the status of soil fertility [12]. Inappropriate N fertilization rates, along with wide variation in sowing windows without considering the impact of rainfall in adopted sowing windows, have impacted upland rice productivity. Rice growth, yield and quality is significantly affected by sowing date [13,14] and sowing is susceptible to impacts of climate change [15]. Thus, the main challenge is to optimize the N input to a level where the crop productivity is not affected for each adopted sowing window. An improved N use efficiency is desired in rice cropping systems. A higher N use efficiency would result in an optimal N fertilization rate for upland rice [4]. Nitrogen utilization patterns also differ under different sowing periods based on variations in moisture availability, which is regulated by rainfall events. Therefore, an optimal N fertilizer application rate tailored to each sowing window will help to maximize N utilization and resource use efficiency, which will enhance the productivity and profitability of upland rice.

Crop simulation models are effective tools that can be utilized to evaluate various crop management options [16] and practices under different environments and seasons [17]. Various models have been used successfully to evaluate different crop management strategies and assessments, i.e., DSSAT [18], EPIC [19], ORYZA [20], CropSyst [21] and APSIM-ORYZA [22]. Models can simulate various aspects of rice-crop growth production, including phenological development, water and nutrient management and environmental impact assessment [23]. The models are unique in their methodological schemes, i.e., some models are efficient in simulating crop growth, considering the water, nutrients and biophysical dynamics [24,25,26,27], while others are constructed to simulate hydrology, pesticide impact and pollutants in rice systems [28,29]. CSM–CERES–Rice is a physiological model present in DSSAT modeling systems, which has been utilized in simulating management scenarios for rice crops. CSM–CERES–Rice has been used to simulate phenology and yield [30,31,32,33], fertilizer management [25,34,35], evaluate climate risks [36] and conduct impact assessments of climate change [37,38,39,40,41] on rice. This model was used to assess N management and determine the economically optimum N application rate [35]. The economic optimum N fertilization rate (EONFR) can be determined using quadratic equations and models [42,43]. However, EOFNR has not been developed to account for the climatic influences caused by sowing windows. In addition, quadratic models do not provide information about the underlying dynamics of nutrients and plant responses while determining the plateau yields and the optimum N fertilization rates. In this regard, crop simulation models can be useful and efficient alternatives for determining EONFR by simulating impacts of N fertilization rates in crop yields, crop N uptake and utilization and N use efficiencies. Puntel et al. [44] employed a calibrated APSIM model to determine optimal N rate for corn by running the APSIM model at 5 kg N ha^−1^ intervals. The DSSAT and APSIM models can be run on desired N rates to observe plateau yield or up to the N rate where the crop no longer responds to N addition, and the economically optimum N fertilization rate can be identified without the use of regression models [44]. This unique characteristic of crop simulation models provides superiority over regression or quadratic equations. 

Insufficient research evidence and recommendations exist for N fertilization rates in upland rice planting across a wide range of sowing dates in Southern Thailand. In this regard, the utilization of CSM–CERES–Rice could be a rapid and cost-effective practical application to determine EONFR for upland rice. According to the authors’ knowledge, the ability of CSM–CERES–Rice has not yet been explored to date to determine sowing-date-specific EONFR. Therefore, the objectives of the study were to: (i) evaluate the performance of CSM–CERES–Rice in simulating upland rice productivity under different N fertilizations and sowing windows, (ii) optimize N fertilization rate according to the adopted sowing windows and (iii) determine the sowing-date-specific EONFR for upland rice.

## 2. Results

### 2.1. Model Calibration and Performance Evaluation

#### 2.1.1. Phenology

**Figure 1:**
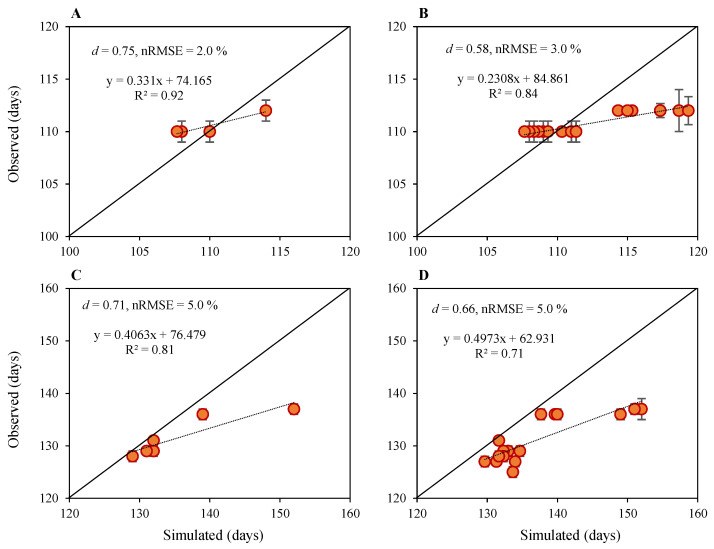
Results of model calibration (**left**) and performance evaluation (**right**) for simulated versus observed days to anthesis (**A**,**B**) and days to maturity (**C**,**D**) after the planting of upland rice planted under four nitrogen fertilization treatments, three sowing dates and two growing seasons. Data points are the average, and vertical error bars represent ± standard errors for observed data obtained from three experimental replicates.

The CERES–Rice model performed well in predicting phenology for all nitrogen fertilization rates (NFRs) and sowing windows in both calibration and evaluation except for the first sowing window (SD1), where the model performance was reasonably good (Figure 1). The difference in the number of days to anthesis for model calibration ranged from −2 to 2 days for N_90_ under SD1, SD2 and SD3 for both seasons’ data, whereas the difference in the number of days to anthesis for model evaluation ranged from −1 to 2 days for SD2 and SD3 for both seasons’ data. For SD1, the difference in the number of days to anthesis for model evaluation ranged from −5 to −7 in the first season, and −2 to −3 in the second season. A similar model performance pattern was observed for simulating days to maturity. Differences in the number of days to maturity for model calibration ranged from −3 to −15 for SD1 and −2 to −3 days for N_90_ under SD2 and SD3 in both seasons, respectively, whereas the difference in the number of days to maturity for model evaluation ranged from −1 to −9 days for SD2 and SD3 in both seasons. For SD1, the difference in the number of days to maturity for model evaluation ranged from −13 to −15 in the first season and from −1 to −4 in the second season.

#### 2.1.2. Grain Yield and Aboveground Dry Matter

**Figure 2:**
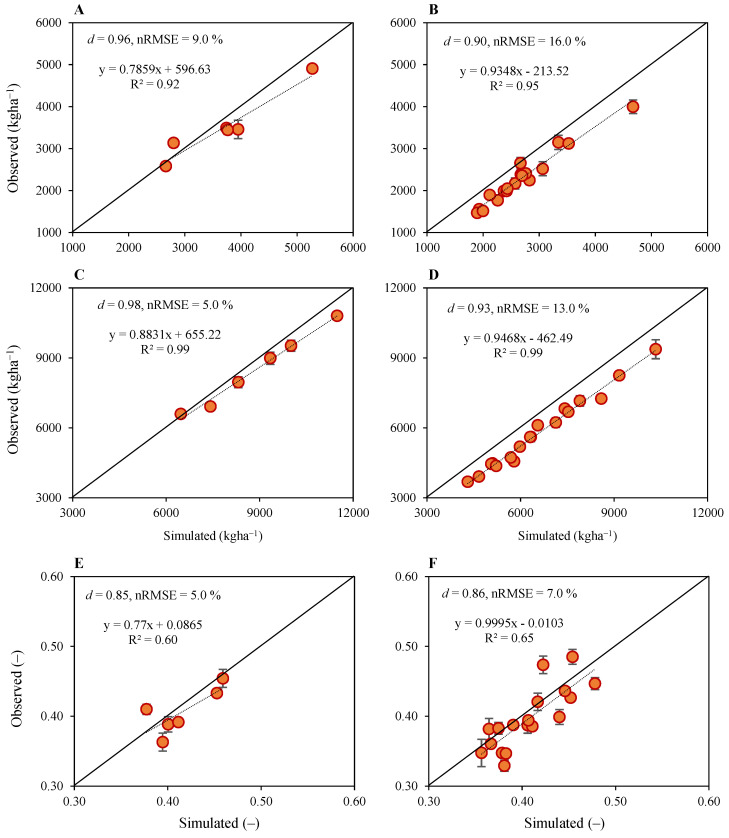
Results of model calibration (**left**) and performance evaluation (**right**) for simulated versus observed grain yield (**A**,**B**), aboveground dry matter (**C**,**D**) and harvest index (**E**,**F**) of upland rice planted under four nitrogen fertilization treatments, three sowing dates and two growing seasons. Data points are the average, and vertical error bars represent ± standard errors for observed data obtained from three experimental replicates.

The CERES–Rice performed well in simulating grain yield, aboveground dry matter and harvest index for all nitrogen fertilization rates (NFRs) (Figure 2) with excellent simulation accuracy. For the grain yield, statistical indicators d-index and nRMSE were 0.96 and 9% for model calibration, respectively, for N_90_ under SD1, SD2 and SD3 for both seasons’ data, and they were 0.90 and 16% for model evaluation, respectively, for N_0_, N_30_ and N_60_ for both seasons’ data (Figure 2A,B). Generally, the grain yield was underestimated except for N_90_ under SD1, where it was overestimated by 11%. The underestimation of grain yield for model calibration with N_90_ under SD1, SD2 and SD3 for both seasons’ data ranged within 3–14%, whereas the underestimation of grain yield for model evaluation with N_0_, N_30_ and N_60_ for SD1, SD2 and SD3 for both seasons ranged 1–32%. Statistical indicators d-index and nRMSE for aboveground dry matter were 0.98 and 5% for model calibration, respectively, for N_90_ under SD1, SD2 and SD3 for both seasons’ data, and they were 0.93 and 13% for model evaluation, respectively, for N_0_, N_30_ and N_60_ for both seasons’ data (Figure 2C,D). Aboveground dry matter was also underestimated in general except for N_90_ under SD3 in the second season, which was overestimated only by 2%. Underestimation of aboveground dry matter for model calibration with N_90_ under SD1, SD2 and SD3 for both seasons’ data ranged within 4–7%, whereas the underestimation of aboveground dry matter for model evaluation with N_0_, N_30_ and N_60_ for SD1, SD2 and SD3 for both seasons ranged within 7–27%. The d-index and nRMSE for aboveground dry matter were 0.85 and 5% for model calibration, respectively, for N_90_ under SD1, SD2 and SD3 for both seasons’ data, and were 0.86 and 7% for model evaluation, respectively, for N_0_, N_30_ and N_60_ for both seasons’ data (Figure 2E,F). Simulations for harvest index also followed a similar trend with grain yield and aboveground dry matter and they were generally underestimated except for all nitrogen treatments in SD1 in the second season, where it was overestimated by 1–11% and for N_30_ in SD2 and SD3 during the first season, where it was overestimated by 5% and 2%, respectively. The underestimation of the harvest index for model calibration with N_90_ under SD1, SD2 and SD3 for both seasons’ data ranged within 1–9%, except for SD1 in the second season, where it was overestimated by 8%. Similarly, an underestimation of the harvest index for model evaluation with N_0_, N_30_ and N_60_ for SD1, SD2 and SD3 for both seasons ranged within 1–16%, except for N_0_, N_30_ and N_60_ in the second season, where it was overestimated by 6%, 1% and 11%, respectively.

#### 2.1.3. Nitrogen Uptake

**Figure 3:**
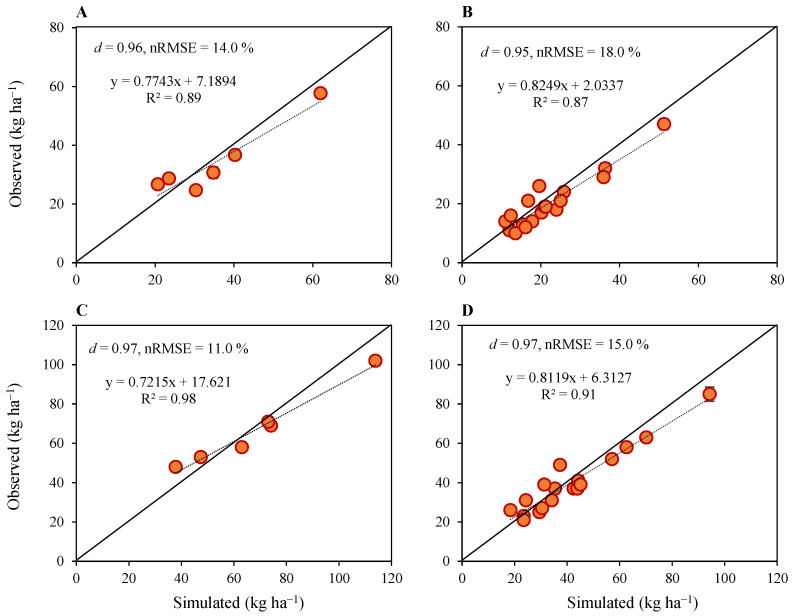
Results of model calibration (**left**) and performance evaluation (**right**) for simulated versus observed grain nitrogen uptake (**A**,**B**), and total crop nitrogen uptake (**C**,**D**) of upland rice planted under four nitrogen fertilization treatments, three sowing dates and two growing seasons. Data points are the average, and vertical error bars represent ± standard errors for observed data obtained from three experimental replicates.

Simulation results for grain and crop nitrogen uptake indicated that the model performed well for both calibration and evaluation (Figure 3). For the grain nitrogen, d-index and nRMSE were 0.96 and 14% for model calibration, respectively, for N_90_ under SD1, SD2 and SD3 for both seasons’ data, and they were 0.95 and 18% for model evaluation, respectively, for N_0_, N_30_ and N_60_ for both seasons’ data (Figure 3A,B). Generally, the grain nitrogen was underestimated, except in the second season, for N_60_ and N_90_ under SD1, where it was overestimated by 25% and 19% and for N_0_, N_30_, N_60_ and N_90_ under SD3, where it was overestimated by 20–23%, respectively. The underestimation of grain nitrogen for model calibration with N_90_ under SD1, SD2 and SD3 for both seasons’ data ranged within 7–21% except for N_90_ under SD1 and SD3, where it was overestimated by 19% and 23%, respectively. Grain nitrogen was underestimated for SD1, SD2 and SD3 for both seasons by 8–33% except for N_60_ under SD1, which was overestimated by 25% and for N_0_, N_30_ and N_60_ under SD3 that were overestimated by 22, 23 and 20%, respectively, in the second season for model evaluation with N_0_, N_30_ and N_60_ nitrogen fertilization treatments.

For the total crop nitrogen uptake, d-index and nRMSE were 0.97 and 11%, respectively, for model calibration for N_90_ under SD1, SD2 and SD3 for both seasons’ data, and they were 0.97 and 15%, respectively, for model evaluation for N_0_, N_30_ and N_60_ under SD1, SD2 and SD3 for both seasons’ data (Figure 3C,D). Simulations for total crop nitrogen uptake also followed an identical trend with grain nitrogen and they were generally underestimated, except for all nitrogen treatments in SD3 in the second season, where it was overestimated by 20–29%, and for N_60_ in SD3 during the first season, where it was overestimated by 4%, and for N_90_ during the second season, where it was overestimated by 11%. The underestimation of total crop nitrogen uptake for model calibration with N_90_ under SD1, SD2 and SD3 for both seasons’ data ranged within 3–19%, except for SD1 in the second season, where it was overestimated by 11%, and for SD3 in the second season, where it was overestimated by 21%. Similarly, the underestimation of total crop nitrogen uptake for model evaluation with N_0_, N_30_ and N_60_ for SD1, SD2 and SD3 for both seasons ranged within 2–18%, except for N_30_ under SD3 in the first season, where it was overestimated by 4%, and for N_60_ under SD1 in the second season, where it was overestimated by 24% and for N_0_, N_30_ and N_60_ in the second season, where it was overestimated by 29%, 21% and 20%, respectively.

#### 2.1.4. Nitrogen Use Efficiencies

**Figure 4:**
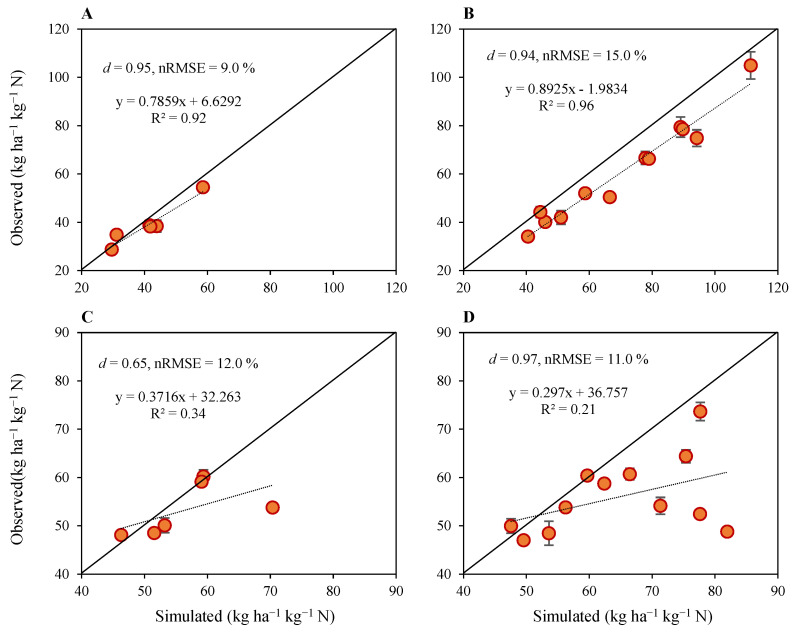
Results of model calibration (**left**) and performance evaluation (**right**) for simulated versus observed nitrogen use efficiency (**A**,**B**), and nitrogen utilization efficiency (**C**,**D**) of upland rice planted under four nitrogen fertilization treatments, three sowing dates and two growing seasons. Data points are the average, and vertical error bars represent ± standard errors for observed data obtained from three experimental replicates.

Model performance for simulating N use efficiencies was also satisfactory for both calibration and evaluation treatments (Figure 4). Generally, the N use efficiencies decreased as N fertilization rates increased, as indicated by simulation results. The d-index and nRMSE for N use efficiency were 0.95 and 9% for model calibration, respectively, for N_90_ under SD1, SD2 and SD3 for both seasons’ data, and they were 0.94 and 15% for model evaluation, respectively, for N_0_, N_30_ and N_60_ for both years of data (Figure 4A,B). In general, the N use efficiencies under N fertilization treatments were underestimated except for N_90_ under SD1 in the second season, where it was overestimated by 11%. The underestimation of N use efficiency for model calibration with N_90_ under SD1, SD2 and SD3 for both seasons’ data ranged within 3–14% except under SD1, where it was overestimated by 11%. Model evaluation results indicated that N use efficiency was underestimated for SD1, SD2 and SD3 in both seasons, ranging from 1 to 32% (Figure 4A,B). 

Compared to N use efficiency, model performance for N utilization efficiency was slightly affected and the resulting d-index and nRMSE values were 0.65 and 12% for model calibration, respectively, for N_90_ under SD1, SD2 and SD3 for both seasons’ data, and they were 0.97 and 11% for model evaluation, respectively, for N_0_, N_30_ and N_60_ under SD1, SD2 and SD3 for both seasons’ data (Figure 4C,D). Simulations for N utilization efficiency also followed a trend similar to that with N use efficiency, and generally NUtE was underestimated, except for N_90_ in SD2 and SD3 in the first year, where it was overestimated by 4% and 1%, respectively, and for N_30_ in SD2 during the second season, where it was overestimated by 1%. There were no differences between simulated and observed values for N_90_ under SD1 in the second season. Underestimation of N utilization efficiency for model calibration with N_90_ under SD1, SD2 and SD3 for both seasons’ data ranged within 6–31%, except under SD2 and SD3 in the first season, where it was overestimated by 4% and 1%, respectively, and no differences were observed for SD1 in the second season. Similarly, the underestimation of N utilization efficiency for model evaluation with N_0_, N_30_ and N_60_ for SD1, SD2 and SD3 ranged from 5 to 17% in the first season and 5 to 68% in the second season, except for N_30_ under SD2, where it was overestimated by 5% and 1%, respectively, in the first and second seasons.

### 2.2. Simulations for Optimum Nitrogen Fertilization Rate

**Figure 5:**
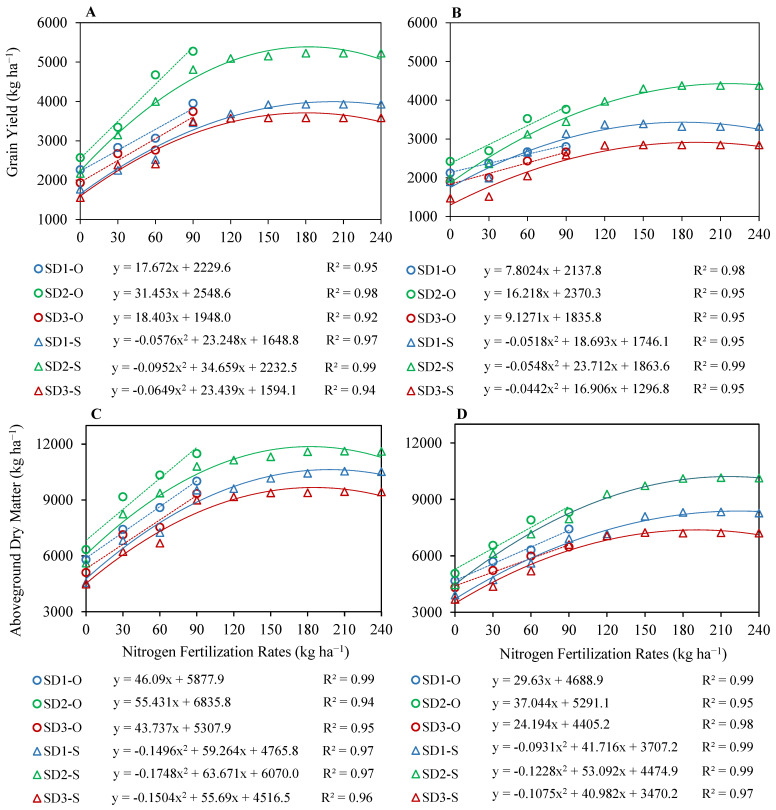
Regression relationships between nitrogen (N) fertilization rates and observed (linear—O) and simulated (quadratic—S) grain yield (**A**,**B**) and between N fertilization rates and observed (linear) and simulated (quadratic) aboveground dry matter (**C**,**D**) for upland rice planted at three sowing dates during the first growing season 2018–2019 (**A**,**C**) and the second growing season 2019–2020 (**B**,**D**).

**Figure 6:**
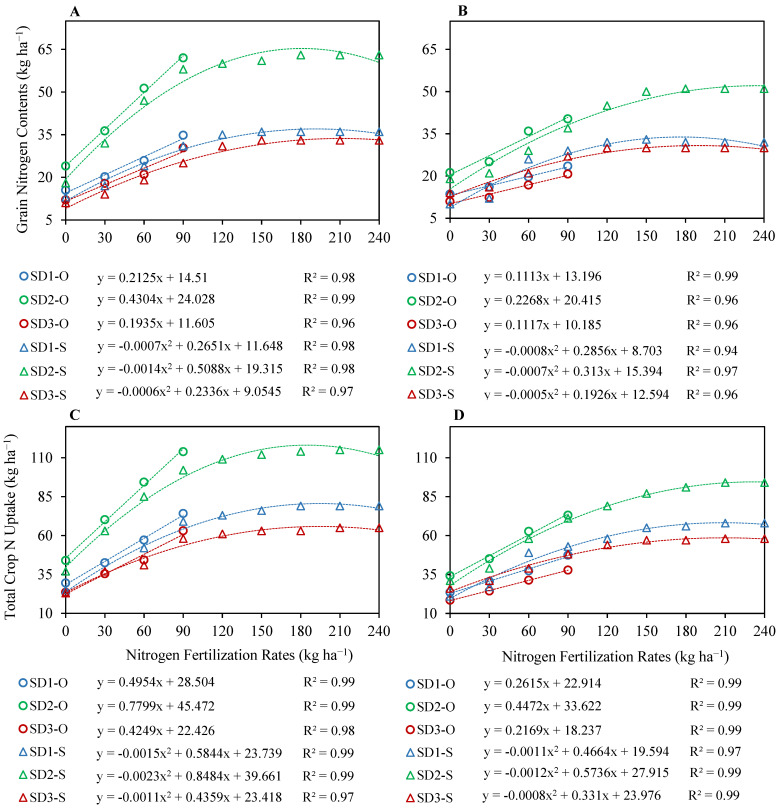
Regression relationships between nitrogen (N) fertilization rates and observed (linear—O) and simulated (quadratic—S) grain N contents (**A**,**B**) and between N fertilization rates and observed (linear) and simulated (quadratic) total crop N uptake (**C**,**D**) for upland rice planted at three sowing dates during the first growing season 2018–2019 (**A**,**C**) and the second growing season 2019–2020 (**B**,**D**).

Observed data under N fertilization with N_0_, N_30_, N_60_ and N_90_ in all sowing windows indicated a linear trend for grain yield and aboveground dry matter (Figure 5) and grain N contents and total crop N uptake (Figure 6). The calibrated CSM–CERES–Rice was then used to simulate the yields by extending the N fertilization rate beyond observed N fertilizer rates at increments of 30 kg up to 240 kg N ha^−1^ (N_120_, N_150_, N_180_, N_210_ and N_240_) until the grain yield no longer responded to N fertilization. By increasing the range of N fertilizer rates, simulated grain yield, dry matter, grain N content and crop N uptake response curves were observed to obey quadratic regression curves with a distinct plateau (Figure 5 and Figure 6). Simulations indicated that the grain yield of upland rice was responsive to N fertilization up to N_180_ under SD1 and SD2, and up to N_150_ under SD3 during the first season; and up to N_150_ under SD1, up to N_180_ under SD2 and up to N_150_ under SD3 during the second season—whereas no further increase in grain yield was observed beyond these N fertilization rates. Grain yield only declined under SD1 in the second season at N_180_ and was constant up to N_240._ Aboveground dry matter indicated a slightly different trend in comparison to the grain yields. It increased up to N_210_ under SD1, SD2 and SD3 during the first season, and up to N_110_ under SD1 and SD2, and up to N_150_ under SD3 during the second season. Aboveground dry matter slightly declined at N_240_ under all sowing windows in both seasons, except for SD3 in the second season, where it was slightly variable between N_150_ and N_240_. 

Like the grain yields, simulations indicated that grain N contents of upland rice were responsive to N fertilization up to N_150_ under SD1 and SD2, and up to N_150_ under SD3 during the first season; and up to N_150_ under SD1, up to N_180_ under SD2 and up to N_120_ under SD3 during the second season. No further increase in grain N contents was observed beyond these N fertilization rates, and they were similar up to N_210_, except under SD1 in the second season, where they decreased by only 1 kg ha^−1^. Total crop N uptake increased with N fertilization rate up to N_180_ under SD1, up to N_210_ under SD2 and SD3 during the first season; and up to N_210_ under SD1, SD2 and SD3 during the second season. No further increase in total crop N uptake was observed beyond N_210_, and total crop N uptake was similar at N_210_ and N_240_.

### 2.3. Optimization of Nitrogen Fertilization Rate for Different Sowing Windows

Optimization of N fertilization rate for different sowing windows was performed by running CSM–CERES–Rice at different N fertilization rates with an increment of 10 kg of applied N below and above the yield plateaus, instead of using the quadratic equations (Figure 5A,B) for simulated grain yields. The model was run to identify the economically optimum nitrogen fertilization rate (EONFR) that resulted in maximum marginal net return (MNR) and profit over control (N_0_). Gross return was increased with N fertilization. Extended simulations to determine the EONFR indicated that the highest profit with N fertilization over N_0_ was observed at N_140_ for SD1, at N_170_ for SD2 and at N_130_ for SD3 during the first growing season; and at N_140_ for SD1, at N_180_ for SD2 and at N_150_ for SD3 during the second growing season. A narrow margin of profit over control, MNR, allowed for the precise estimation of EONFR. The highest MNR was observed at N_140_ for SD1, at N_170_ for SD2 and at N_130_ for SD3 for both seasons (Table 1). The highest profit and MNR were observed at SD2 in both seasons. Simulated grain N contents, total crop N uptake and N use efficiencies were also at their highest under SD2 (Table 2). Based on grain yield performance, the highest MNR and promising levels of grain N contents, total crop N uptake and N use efficiencies, N_140_ for SD1, N_170_ for SD2 and N_130_ for SD3, respectively, were identified as EONFR.

## 3. Discussion

Optimal nitrogen (N) management according to adopted sowing windows is critical to achieve viable productivity, N use efficiency and profitability of upland rice. An adequate amount of N availability is highly important in rice production, as N is an essential element for rice growth. Nitrogen utilization is also affected by the effect of adopted sowing windows due to climatic conditions and particularly the moisture availability. An overapplication of N results in higher N losses into the environment and reduces profitability, whereas the underapplication of N results in agronomic losses and reduced rice growth and productivity. Various methods and tools are used to determine plant N requirement, and each has its own limitation on providing the desired information. Process-based crop models provide rapid and cost-effective means to determine plant N requirements in this scenario. In this study, we evaluated the performance of CSM–CERES–Rice to simulate upland rice responses to N fertilization treatments under different sowing windows. The model was further used to optimize the N fertilization rate for upland rice according to adopted sowing windows. 

The phenology of upland rice indicated a similar trend for days to anthesis and days to maturity between simulated and observed data in respective sowing windows. The model also performed well in predicting the phenology for different sowing windows. Other studies simulating the phenology of rice have indicated that CSM–CERES–Rice was efficient in predicting phenological responses of rice [26,45,46]. Model performance was reasonable at simulating phenology for SD1, particularly in the first season. This was because of a longer period for the collection of observed data for SD1 relative to later sowing dates. Lu et al. [47] also observed that the performance of CSM–CERES–Rice was affected in simulating phenology under some treatments, i.e., the difference in the number of days to flowering and maturity for late rice was 7 and 9 days, respectively. Maximum crop duration was observed in SD1, and the shortest crop duration was observed in SD3. Although the model was responsive to adjustment in phenology coefficients and simulation accuracy could be improved for SD1; however, further adjustments for phonology coefficients were avoided to improve performance in SD2 and SD3 in order to maintain accuracy in other crop responses, i.e., grain yield and crop N uptake. Therefore, phenology coefficients were adjusted to the best performance of maximum attributes in all sowing windows. Crop duration was the longest in SD1 and was the shortest in SD3 because of extended and shortened vegetative phases in SD1 and SD3, respectively, caused by temperature and rainfall differences as discussed earlier [48]. It is well known that plant growth and development are affected by the effect of changes in high and low temperatures [49]. Similarly, rice growth duration is sensitive to changes in temperature and rainfall, which can be delayed with low temperatures and rainfall and can be shortened at high temperatures [50].

The results indicated that the model was sensitive to N addition, and upland rice productivity increased with N fertilization rate. Nitrogen fertilization enhanced grain yield and aboveground dry matter production and also improved the harvest index. Increases in grain yield, aboveground dry matter production and harvest index of rice with N rate are well documented [51,52,53]. CSM–CERES–Rice performance at predicting grain yield and aboveground dry matter was excellent with high values for *d*-index and low values for nRMSE. The *d*-index and nRMSE values for harvest index were comparatively low compared to the values for grain yield and aboveground dry matter. This was possibly because the simulated harvest index was computed from simulated grain yield and aboveground dry matter, and there was a gap between the simulated and observed grain yield and aboveground dry matter. However, there was still an excellent agreement between simulated and observed harvest index, with nRMSE less than 10%. Previous studies have indicated that CSM–CERES–Rice was sensitive to N addition, and was able to predict yields under different N fertilization rates [46,54] and different management options [35,47,54]. In our study, generally, the grain yields and aboveground dry matter were underestimated. These results were in line with the findings of Lu et al. [47] who also observed that CSM–CERES–Rice underestimated the yields under different N application and management. 

Nitrogen fertilization enhanced grain N contents and total crop N uptake as a result of an increased N supply. Ullah et al. [55] stated that plant performance is determined by the interaction between plant N uptake and N losses, whereas a high plant N uptake contributes to a higher biomass productivity. Proper N management also enhances plant N uptake and utilization. Numerous studies have indicated that rice crop N uptake is enhanced under an increased N supply [53,55]. CSM–CERES–Rice showed sensitivity for N uptake with N additions and was able to predict grain and total crop N uptake under different N fertilization rates. A previous study also indicated that N uptake predictions using CSM–CERES–Rice increased with N addition and different management options in rice [46]. Like the grain yield and aboveground dry matter, the grain N contents and total crop N uptake were underestimated in general. The model performance for estimating grain N contents and total crop N uptake was slightly lower, with nRMSE values ranging between 11 and 18% as compared to simulation accuracy for grain yield and aboveground dry matter, where the nRMSE ranged between 5 and 16%. This was in line with the findings of Kadiyala et al. [46], who found that the performance of CSM–CERES–Rice based on RMSE and nRMSE was lower when simulating grain and total crop N uptake as compared to estimating grain yields. 

Nitrogen use efficiencies decreased with increased N fertilization. Generally, N use efficiency is decreased with an increased N supply in rice due to an increased supply of N in soil [56]. Santiago-Arenas et al. [56] stated that N use efficiency in rice decreased with increases in N fertilization rate, and similar results were reported by Sung et al. [57] and Alou et al. [58]. The simulation accuracy of CSM–CERES–Rice for N use efficiencies was also similar to the performance for simulating N uptake, with nRMSE values ranging between 9 and 15%. Nitrogen use efficiencies were useful indicators for evaluating the impact of sowing windows. The N use efficiencies varied greatly among sowing windows both for simulated and observed data, in addition to simulations for expanded N fertilization rates in respective sowing windows. Thus, N use efficiencies can be used to identify the most suitable sowing window in upland rice management. 

With the identification of a suitable sowing window, sowing-date-specific economic optimal N fertilization rate (EONFR) is a valuable consideration. Our results in this study indicate that adjusting N fertilization rates according to the different sowing windows is beneficial, compared to the general recommendation of a similar N fertilization rate for all sowing windows. We observed that grain yield and upland rice performance could be non-optimal with a uniform N fertilization rate across sowing windows. This was due to the climatic variations that prevailed during each sowing window. A previous study confirmed that rainfall distribution and temperature were highly variable among three sowing windows [48]. Hence, a crop growth modeling approach is a practical approach for determining an optimal N fertilization management strategy, which is customized for field information and weather data. Using this approach, farmers can be advised and EONFR can be recommended for different sowing windows. However, a challenge is that the model results are highly dependent upon the quality of input data, particularly the soil and weather data. Detailed soil and precise weather data are necessary for efficient model predictions as soil and weather are highly variable in upland regions. In this regard, the meteorological department and rice department should work closely with research institutions and farmers to provide desired information for the recommendation of N fertilization rates for different zones.

In short, sowing-date-specific EONFR determined in this study provides a useful N fertilization management option for upland rice by utilizing the critical information on plant N uptake and N utilization. CSM–CERES–Rice can be used to determine the sowing-date-specific EONFR based on provided soil and weather conditions. We also recommend for further studies to classify upland rice zones based on soil variability and weather patterns. A highly efficient soil database and accurate weather prediction system would provide precise soil fertility status and weather information, respectively, enabling the model to provide a more efficient estimation of EONFR.

## 4. Materials and Methods

### 4.1. Study Area

The experimental site (7°00′14.5″ N, 100°30′14.7″ E) is situated in Songkla province, which is in the eastern part of Southern Thailand, where mean maximum and mean minimum annual temperature reaches 32.1 °C and 24.2 °C, respectively, with an average annual rainfall of 2521.4 mm [59]. The climate of Southern Thailand is characterized by hot or dry season and rainy season [4]. Generally, considering the annual cycle of precipitation over most of Thailand, the rainy season lasts from May to October, whereas the dry season lasts from November to April of the following year [10,60]. However, the climate of Southern Thailand is highly variable [4]. Therefore, the classification of the rainy season occurring from May to October does not apply in the eastern part of Southern Thailand, and most of the precipitation in this part occurs from November to February of the following year [10].

### 4.2. Field Experiments and Management

Field experiments were conducted at a field research area of the Faculty of Natural Resources, Prince of Songkla University, in Songkla province of Thailand during 2018–2019 and 2019–2020. *Dawk Pa–yawm* which is a commonly cultivated cultivar in Southern Thailand was used in this assessment. Soil sampling was performed at three soil depths of 0–30, 30–60 and 60–120 cm prior to planting, and soil was analyzed for soil properties such as texture, water characteristics, bulk density, pH, organic carbon, total nitrogen, nitrate—N and available phosphorus and potassium. Observed soil properties are presented in Table S1. Experiments were conducted with three nitrogen fertilization rates, including 30 kg N ha^−1^ (N_30_), 60 kg N ha^−1^ (N_60_) and 90 kg N ha^−1^ (N_90_), as well as a control with no N fertilization (N_0_), and planting was performed at three sowing dates (SD): SD1 (August/September), SD2 (September/October) and SD3 (October/November). Prior to planting in both seasons, all experimental plots were equally fertilized with phosphorus (19 kg P_2_O_5_ ha^−1^) and potassium (13 kg K_2_O ha^−1^) [48]. Nitrogen fertilization was performed in two splits at tillering and panicle initiation stages using urea (46% N) as a N fertilizer source. Supplementary irrigation was also applied in experimental plots at planting and during the hot and dry intervals of each sowing window. All the treatments were repeated three times. Detailed information on field preparation, experimental design and plot management, basal fertilization, N fertilizer treatments application, supplementary irrigation and insect pest and weed management can be accessed in our previous study [48]. 

### 4.3. Data Collection and Computations

Crop data collected included crop phenology, yield and yield components, recorded for all treatments. Soil and plant sampling was also performed in all experimental plots to observe N concentrations and determine N contents. Nitrogen uptake was computed by multiplying the respective grain and straw yields with N concentrations. Detailed procedures on the measurement of the crop data as well as soil properties and determination of N data for experimental site can be referred to in our previous work [48]. The N utilization efficiency (NUtE: kg ha^−1^ kg^−1^ N) (1) and N use efficiency (NUE: kg ha^−1^ kg^−1^ N) (2) [34] were computed using the following equations:

(1) $$NUtE=\frac{grain\;yield\;(N_{x})}{N\;uptake\;(N_{x})}$$

(2) $$NUE=\frac{grain\;yield\;(N_{x})}{N\;applied\;(N_{x})}$$

Meteorological data required for model input were collected from Agrometeorology–Agricultural Information Center of Kho Hong, Hat Yai (7°01′06.0″ N, 100°29′52.1″ E), located 1.8 km from the experimental site. Meteorological data used in simulation study are available online and can be accessed freely through the Automatic Weather System of the Thai Meteorological Department (AWS–TMD) (http://www.aws-observation.tmd.go.th/main/main, accessed on 19 August 2021).

### 4.4. Model Configuration and Simulations

Input files, including FileX, Soil file and Weather file, were created using the XBuild, SBuild and WeatherMan modules, respectively, in CSM–CERES–Rice with the collected soil, crop, and weather data. Soil properties were specified in SBuild and soil profile was created. Daily weather data were used to create weather station using WeatherMan tool present in the DSSAT–*v*4.8.0. Field conditions, crop and crop management operations, including rice cultivar information, planting details, irrigation method and applications, fertilizer applications and method and tillage and harvest information, were specified in the management file using XBuild. Model calculation methods and controls, including simulation information, water and N modules, soil–water balance, weather, evapotranspiration, photosynthesis, hydrology, management, etc., were specified in the simulation option section of XBuild. Generalized Likelihood Uncertainty Estimation (GLUE) tool [61,62] present in the DSSAT–*v*4.8.0, which is a Bayesian estimation method and has been widely used in modeling studies [62], was used to readjust previously determined cultivar coefficients for upland rice genotype (*cv*: *Dawk Pa–yawm*) and the model was calibrated using the dataset observed from highest N fertilization treatment (N_90_) at three sowing windows in both seasons. Cultivar coefficients for plant growth and development were further adjusted in a sequence using trial and error method until a best fit was achieved between the simulated and observed [34]. 

Model evaluation was performed using the dataset from remaining N treatments in three sowing windows in both seasons. Simulated and observed data of days to anthesis, days to maturity, grain yield, aboveground dry matter, harvest index, grain N contents, total crop N uptake and N use efficiencies were compared for model performance. Following model performance evaluation with N fertilization rates of N_0_, N_30_, N_60_ and N_90_, simulations to observe upland rice response to N additions were performed by increasing the N fertilization rate at regular increments of 30 kg up to 240 kg N ha^−1^ (N_120_, N_150_, N_180_, N_210_ and N_240_) until the grain yield no longer responded to N fertilization. To determine the optimal N fertilization rate, the model was run with 10 kg N ha^−1^ increments for each sowing window with the N fertilization rates below and above the points where the highest yield was observed, following the technique mentioned by Puntel et al. [44], by which the economic optimum N fertilization rate (EONFR) can be determined. 

### 4.5. Statistics and Economics

Predicted and observed parameters were compared and model performance was evaluated based on the strength of the statistical indicators, including percentage differences, normalized root-mean-squared error (nRMSE) (3) [63], RMSE (4) and index of the agreement (*d*) (5) [64] using the following equations. Positive and negative values of percentage differences indicate an overestimation and underestimation of the results, respectively. A low nRMSE value and high *d*-index value near to 1 is an indication of a higher simulation accuracy and vice versa. Coefficient of multiple determinations (R^2^) was used in regression analysis. A high R^2^ value is indicative of the accuracy of the results.

(3) $$\mathrm{nRMSE}=\frac{\mathrm{RMSE}\times 100}{\bar{O}}$$

(4) $$\mathrm{RMSE}=\sqrt{\frac{\;\sum_{i=1}^{n}(P_{i}-O_{i}{)}^{2}}{n}}$$

where, *P_i_* refers to the model predicted value, *O_i_* refers to the observed field value, *n* refers to the number of observations and *Ō* refers to the overall mean of the observed field values.

(5) $$d=1-\frac{\sum_{i=1}^{n}(P_{i}-O_{i}{)}^{2}}{\sum_{i=1}^{n}(\left|P_{i}^{\prime }\right|+\left|O_{i}^{\prime }\right|{)}^{2}}$$

where, *n* refers to the number of observations, *P_i_* refers to the model predicted value, *O_i_* refers to the observed field value, $P_{i}^{\prime }$ is *P_i_* − *Ō* and $O_{i}^{\prime }$ is *O_i_* − *Ō*.

Profitability for grain yield in response to N fertilization was computed for the dataset observed from the experiments in addition to the simulated yields for additional sites. Economic assessment was performed and gross returns and profits upon N fertilization for each simulated treatment were computed based on the urea fertilizer cost (THB 800 = USD 23.76 per 50 kg bag) and rice grain (*Dawk Pa–yawm*) selling price (THB 60 = USD 1.78 kg^−1^) in the year 2018 [48]. The EONFR was estimated based on computed marginal net return (MNR) (6) [35]. The EONFR for a treatment was considered where the maximum MNR was observed:

(6) $$Marginal\;Net\;Return\left(MNR\right)=Grain\;yield\times Price-NFR\times Nc$$

where, the grain yield is simulated grain yield with N fertilization, Price is the selling price of rice (USD 1.78 kg^−1^), NFR is the N fertilization rate (kg ha^−1^) and Nc is the cost of N (USD 0.48 kg^−1^).

## 5. Conclusions

The crop simulation model CERES–Rice performed well in simulating phenology, grain yield, aboveground dry matter, grain, total crop N uptake and N use efficiencies for upland rice under different N fertilization rates and sowing windows based on statistical indicators, including *d*-index (approaching to 1) and nRMSE (<30). Simulations for N optimization with extended N fertilization rates up to 240 kg N ha^−1^ (N_240_) indicated that upland rice yield was responsive to N fertilization up to 180 kg N ha^−1^ (N_180_), at which the yield plateau was observed, and no increase in grain yield was observed beyond N_180_. Considering the impact of sowing windows, maximum crop performance for productivity, N uptake, N utilization, profitability and MNR were observed at SD2 in both seasons. Fertilization rates of 140, 170 and 130 kg N ha^−1^ were identified as the economic optimum N fertilization rates (EONFR) for SD1, SD2 and SD3, respectively, based on the computed profitability, marginal net returns and N utilization. Simulation results suggest that the optimum N fertilizer application is a practical management strategy that would enhance the crop productivity, NUE and profitability of upland rice systems. However, it should be noted that climatic conditions, particularly rainfall patterns, are variable over different sowing windows and seasons. In addition, model results also indicated variability in optimum N fertilization rates for different sowing windows. Therefore, N fertilization rates should be determined and practiced according to the prevailing or predicted climatic conditions for different sowing windows and seasons. In this regard, based on the performance of CSM–CERES–Rice, it is recommended that the model be used to determine N fertilization rates for adopted seasonal sowing windows, which will lead to a more efficient N utilization and improved profitability of upland rice production.

## Figures and Tables

**Table 1:** Simulated economic optimum nitrogen fertilization rate (EONFR), grain yields, gross returns, profits over control and marginal net return (MNR) for upland rice planted at three sowing windows (SD1–SD3) during the first growing season 2018–2019 and the second growing season 2019–2020.

Growing Season	Sowing Window	EONFR	Grain Yield	Gross Return	Profit	MNR
		kg ha^−1^	kg ha^−1^	USD ha^−1^	USD ha^−1^	USD ha^−1^
2018–2019	SD1	140	3935.0	7004.0	3859.0	6937.1
	SD2	170	5226.0	9302.3	5437.9	9220.7
	SD3	130	3580.0	6372.4	3602.1	6310.0
2019–2020	SD1	140	3391.0	6036.0	2662.9	5968.8
	SD2	170	4378.0	7792.8	4252.4	7711.2
	SD3	130	2840.0	5055.2	2438.6	4992.8

**Table 2:** Simulated economic optimum nitrogen fertilization rate (EONFR), grain nitrogen (N) contents, total crop N uptake, N use efficiency (NUE) and N utilization efficiency (NUtE) for upland rice planted at three sowing windows (SD1–SD3) during the first growing season 2018–2019 and the second growing season: 2019–2020.

Growing Season	Sowing Window	EONFR	Grain N Contents	Total Crop N Uptake	NUE	NUtE
		kg ha^−1^	kg ha^−1^	kg ha^−1^	kg ha^−1^ kg^−1^ N	kg ha^−1^ kg^−1^ N
2018–2019	SD1	140	36.0	75.0	28.1	52.5
	SD2	170	63.0	114.0	30.7	45.8
	SD3	130	32.0	63.0	27.5	56.8
2019–2020	SD1	140	33.0	64.0	24.2	53.0
	SD2	170	51.0	91.0	25.8	48.1
	SD3	130	30.0	57.0	21.9	49.8

## Data Availability

The original contributions presented in the study are included in the article and Supplementary Materials; further inquiries can be directed to the first and corresponding author.

## Supplementary Materials

The following supporting information can be downloaded at: https://www.mdpi.com/article/10.3390/plants12213685/s1, Table S1. Initial soil parameters at three soil layers of the experimental site (Hat Yai: Songkhla province) used as model input.
