# Supplementary material for: Optimizing Nitrogen Fertilization to Enhance Productivity and Profitability of Upland Rice Using CSM–CERES–Rice
Source: Plants (Basel). 2023 Oct 25;12(21):3685. doi: 10.3390/plants12213685 (PMC10647420; doi:10.3390/plants12213685)
Supplement: Supplementary file 1 [file plants-12-03685-s001.zip › plants-2596686-supplementary.docx]

Supplementary Tables

**Supplementary Table 1.** Initial soil parameters at three soil layers of experimental site (Hat Yai: Songkhla province) used as model input.

| Soil Properties | Soil layer depths (cm) | | |
| --- | --- | --- | --- |
|  | 0−30 | 30−60 | 60−120 |
| Soil texture class | Sandy clay loam | Sandy clay | Clay |
| Sand (%) | 63.76 | 52.30 | 34.27 |
| Silt (%) | 10.48 | 8.93 | 12.98 |
| Clay (%) | 25.76 | 38.77 | 52.75 |
| Field capacity (m^3^m^−3^) | 0.24 | 0.24 | 0.25 |
| Wilting point (m^3^m^−3^) | 0.13 | 0.13 | 0.13 |
| Bulk density | 1.54 | 1.55 | 1.60 |
| Hydraulic conductivity (mmhr^−1^) | 48.0 | 45.0 | 39.0 |
| Soil pH | 5.63 | 5.11 | 5.01 |
| Soil organic matter (gkg^−1^) | 7.43 | 8.21 | 5.59 |
| Soil organic carbon (%) | 0.46 | 0.35 | 0.26 |
| Total nitrogen (gkg^−1^) | 0.47 | 0.43 | 0.37 |
| Initial nitrate (ppm) | 4.50 | 2.40 | 1.20 |
| Available phosphorus (mgkg^−1^) | 40.29 | 23.25 | 9.40 |
| Available potassium (mgkg^−1^) | 53.32 | 70.82 | 73.80 |
